# Incidence of intraspinal abnormalities in congenital scoliosis: a systematic review and meta-analysis

**DOI:** 10.1186/s13018-020-02015-8

**Published:** 2020-10-21

**Authors:** Xudong Wang, Yangke Yu, Ningning Yang, Lei Xia

**Affiliations:** Department of Orthopaedic Surgery, The First Affiliated Hospital of Zhengzhou University, Zhengzhou, 450052 Henan People’s Republic of China

**Keywords:** Congenital scoliosis, Intraspinal abnormalities, Magnetic resonance imaging, Meta-analysis

## Abstract

**Objective:**

This is the first systematic review and meta-analysis on the overall incidence of intraspinal abnormalities in patients with congenital scoliosis (CS) and potential influencing factors.

**Methods:**

We searched three large electronic databases (PubMed, EMBASE, and Cochrane Library) for potentially relevant studies. The quality of the included studies was assessed independently by two authors using the Methodological Index for Non-Randomized Studies (MINORS) criteria. Data on the number of CS patients, number of CS patients with intraspinal abnormalities, sex of the patients, and CS types were extracted from the included studies. R software was used to pool and analyze all the extracted data.

**Results:**

This meta-analysis included 10 articles, and 671 of 1863 CS patients undergoing magnetic resonance imaging (MRI) examinations were identified to have intraspinal abnormalities. The overall incidence of intraspinal abnormalities in the patients with CS was 37% (95% CI, 29–45%). Diastematomyelia was the most common intraspinal abnormality and was detected in 45.60% of the patients with intraspinal abnormalities (306/671). The remaining intraspinal abnormalities included syringomyelia (273/671, 40.69%), tethered cord (190/671, 28.32%), low conus (58/671, 8.64%), intraspinal mass (39/671, 5.81%), Chiari malformation (32/671, 4.77%), fatty filum (27/671, 4.02%), spina bifida (occulta excluded) (17/671, 2.53%), tumor (17/671, 2.53%), cyst (12/671, 1.79%), syringomyelus (4/671, 0.60%), dural ectasia (1/671, 0.15%), and undiagnosed cord MRI hyperintensity (1/671, 0.15%). The patient’s sex and CS type were not factors that affected the incidence of intraspinal abnormalities in CS patients (all *P* > 0.05).

**Conclusions:**

This meta-analysis revealed that the overall incidence of intraspinal abnormalities detected by MRI in CS patients was 37%. Diastematomyelia was the most common intraspinal abnormality. The patient’s sex and CS type were not factors that affected the incidence of intraspinal abnormalities in CS patients.

## Introduction

Congenital scoliosis (CS) is a curvature of the spine resulting from abnormal vertebral development during 4 to 6 weeks of gestation [1, 2]. The incidence of CS in the general population is approximately 1/1000 to 1/2000, and CS is greatly affected by environmental and genetic factors [3–5]. Vertebral anomalies leading to CS are classified according to whether there is failure of formation, failure of segmentation, or mixed abnormalities [6]. The embryonic development of vertebrae is closely related to the development of the spinal cord and organs from the mesoderm [7]. As a result, CS is often associated with intraspinal abnormalities and defects of other organs [1, 8–11]. During the surgical correction of scoliosis, the risk of neurological injury considerably increases with the presence of intraspinal abnormalities [7, 12–14]. It is necessary to detect intraspinal abnormalities in patients with CS before corrective surgery is performed.

For decades, the incidence of intraspinal abnormalities in patients with CS has been noticeable. In 1984, McMaster found intraspinal abnormalities in 18% of 251 patients with myelography [15]. Currently, magnetic resonance imaging (MRI) is considered more sensitive and noninvasive in detecting intraspinal abnormalities [16–18]. Its use has been suggested to comprehensively evaluate CS patients before surgical interventions are performed [19, 20]. However, the incidence of intraspinal abnormalities detected by MRI is highly variable, ranging from 21.8 to 53.7% [1, 16, 19–28]. In addition, it is not known whether sex and CS types affect the incidence of intraspinal abnormalities in patients with CS. Shen et al. [19] found that the incidence of intraspinal abnormalities in patients with a failure of segmentation or mixed abnormalities was significantly higher than that in patients with a failure of formation. Liu et al. [1] reported that there is no significant difference in the incidence of intraspinal abnormalities among patients with different CS types, but the incidence of intraspinal abnormalities was found to be higher in females than in males. Ghandhari et al. [24] showed that there is no significant difference in the incidence of intraspinal abnormalities between males and females.

Although many studies have reported the incidence of intraspinal abnormalities in CS patients, no meta-analyses or systematic reviews have pooled seemingly disparate incidence values to determine the risk of intraspinal abnormalities in CS patients. Therefore, this study was performed to investigate the overall incidence of intraspinal abnormalities in patients with CS and potential influencing factors.

## Methods

### Search strategies and selection criteria

We thoroughly searched three large electronic databases (PubMed, EMBASE, and Cochrane Library) up to October 2019 using the following keywords: “congenital”, “scoliosis”, “magnetic”, “resonance”, “imaging”, and “MRI”. All articles we retrieved from the three databases were published from 1988 to 2019. Two authors (Wang, Yu) independently screened all potentially relevant titles and abstracts, and any disagreements were resolved by a third author.

Studies were included if they (1) included patients with a diagnosis of CS, (2) included more than 30 patients with CS, and (3) detected intraspinal abnormalities by MRI of the whole spine. The exclusion criteria were as follows: (1) no specific intraspinal abnormalities were reported; (2) duplicate data were reported by the same author in the same institution; (3) the article was not published in English; (4) the articles were conference abstracts, animal trials, or a case report; and (5) the patients had metabolic diseases, adolescent idiopathic scoliosis, degenerative scoliosis, simple kyphosis, mixed deformity, or scoliosis secondary to infection or tuberculosis.

### Data extraction

Two authors independently extracted data from the included articles. The following information were extracted: (1) the name of the author, (2) the publication year, (3) the number of patients with CS, (4) the number of CS patients with intraspinal abnormalities, (5) the sex of the patients, (6) the CS types, and (7) any manifestations of intraspinal abnormalities.

### Quality assessment

The quality of the included studies was assessed independently by two authors using the Methodological Index for Non-Randomized Studies (MINORS) criteria [29]. It was established to evaluate the quality of comparative and noncomparative studies; the maximum score is 24 for comparative studies and 16 for noncomparative studies. For noncomparative studies, scores of 0–4 corresponded to very low quality, 5–7 corresponded to low quality, 8–12 corresponded to fair quality, and ≥ 13 corresponded to high quality. For comparative studies, scores of 0–6 corresponded to very low quality, 7–10 corresponded to low quality, 11–15 corresponded to fair quality, and ≥ 16 corresponded to high quality.

### Statistical analysis

R software (version 3.6.1; http://www.r-project.org) was used to pool and analyze all the extracted data from the selected studies. A chi-squared-based Q statistical test was used to determine the statistical heterogeneity among the studies. The degree of heterogeneity for each included study was quantified using the *I*^2^ statistic. When *P* > 0.1 and/or *I*^2^ < 50%, the heterogeneity was considered to be low, and a fixed-effects model was used to generate a forest plot. Otherwise, a random-effects model was used for the meta-analysis. Initially, we calculated the point incidence of intraspinal anomalies in patients with CS and its 95% confidence interval (CI) for each study. Afterwards, the pooled incidence and 95% CI were calculated. The overlap of the 95% CIs showed no differences at the *P* < 0.05 level.

## Results

### Study selection and characteristics

**Fig. 1:**
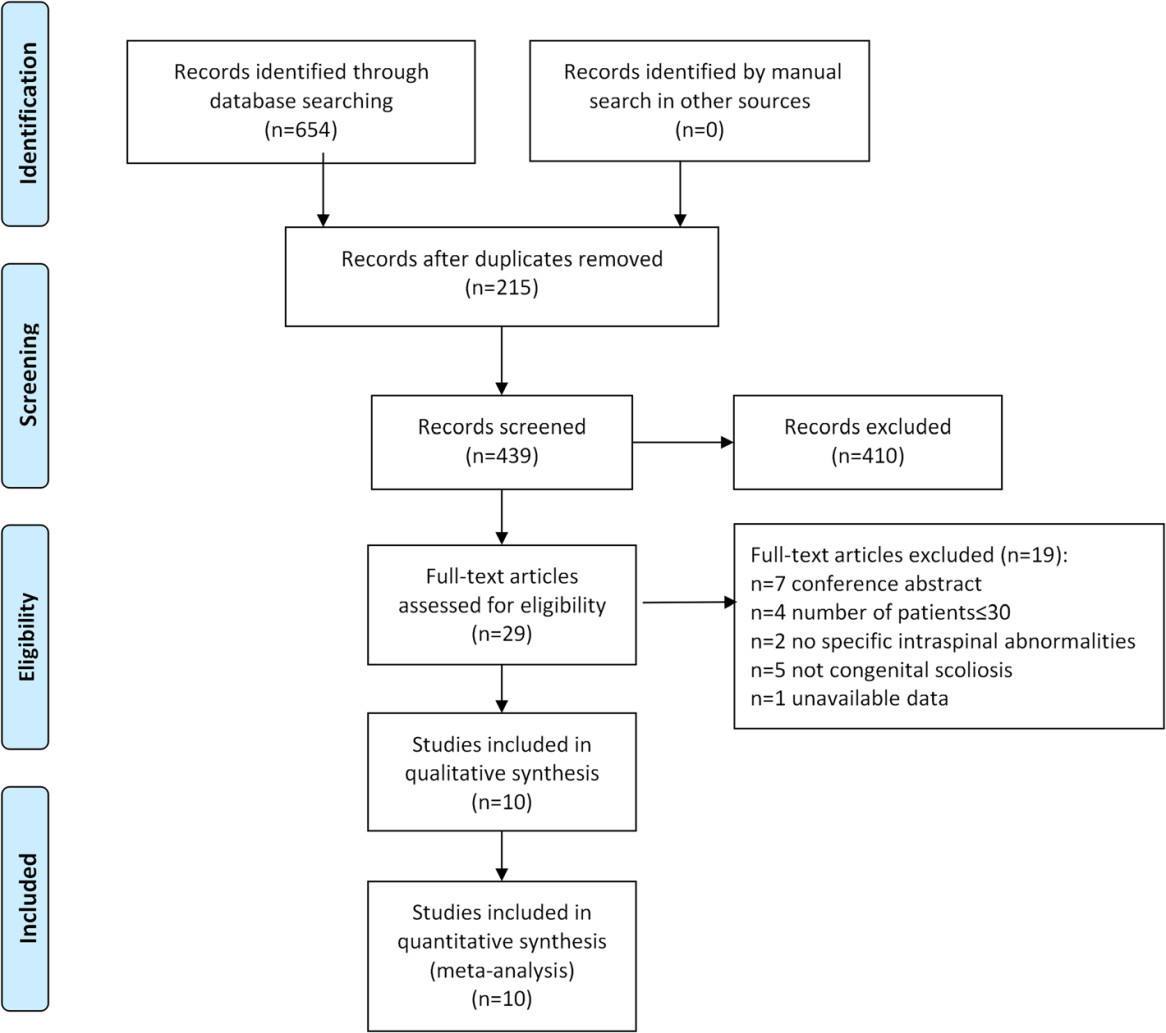
Flow diagram of the study selection process

Initially, 654 potentially relevant studies were identified from three large electronic databases. A total of 215 studies were removed because of overlap. Then, 410 articles were excluded after the titles and abstracts were screened. After the full-text articles were assessed, a total of 10 studies were included in the present study. A flow diagram of the literature selection process is shown in Fig. 1. A total of 10 studies published from 2001 to 2019 described intraspinal abnormalities in CS patients undergoing MRI examinations [1, 16, 19–25, 28]. The basic characteristics of the included studies are shown in Table 1.

**Table 1:** Basic characteristics of included studies

No.	Author	Year	Mean age (year)	CS patients with intraspinal abnormalities/CS patients	Sex		CS types			MINORS
					Male	Female	Failure of formation	Failure of segmentation	Mixed abnormalities	
1	Belmont	2004	4.9	18/76	NR	NR	NR	NR	NR	12
2	Erfani	2007	NR	19/46	NR	NR	NR	NR	NR	8
3	Furdock	2019	NR	113/267	NR	NR	NR	NR	NR	8
4	Ghandhari	2015	13.5	44/202	16/90	28/112	28/108	10/64	6/30	17
5	Gupta	2016	5.3	56/119	16/44	40/75	21/61	15/23	20/35	8
6	Hou	2018	12.25	54/120	NR	NR	NR	NR	NR	13
7	Liu	2011	12.8	132/539	37/214	95/325	71/301	45/168	16/70	8
8	Sevencan	2019	12.6	124/231	43/94	81/137	65/129	41/64	18/38	8
9	Shen	2013	13.9	99/226	33/94	66/132	27/96	29/48	43/82	8
10	Suh	2001	NR	12/37	6/17	6/20	5/19	1/4	6/14	8

*No*. number, *CS* congenital scoliosis, *MINORS* Methodological Index for Non-Randomized Studies, *NR* not reported

### Quality and bias assessment

Two authors evaluated the quality of the included studies independently by using the MINOR criteria. For 8 noncomparative studies, the average MINORS score was 8.5 (range from 8 to 12), suggesting fair quality. The other two comparative studies had scores of 13 and 17, suggesting fair and high quality, respectively.

### Incidence of intraspinal abnormalities

**Fig. 2:**
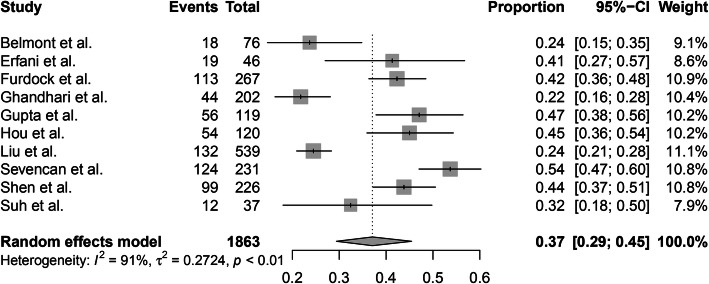
The overall incidence of intraspinal abnormalities in patients with congenital scoliosis

This meta-analysis included 10 articles, and 671 of the 1863 CS patients undergoing MRI examinations were identified to have intraspinal abnormalities. A random-effects model was used due to the high level of heterogeneity (*I*^2^ = 91%). The overall incidence of intraspinal abnormalities in patients with CS was 37% (95% CI, 29–45%) (Fig. 2).

Diastematomyelia was the most common intraspinal abnormality and was detected in 45.60% of patients with intraspinal abnormalities (306/671). The second and third most common intraspinal abnormalities were syringomyelia (273/671, 40.69%) and tethered cord (190/671, 28.32%), respectively. The remaining intraspinal abnormalities included low conus (58/671, 8.64%), intraspinal mass (39/671, 5.81%), Chiari malformation (32/671, 4.77%), fatty filum (27/671, 4.02%), spina bifida (occulta excluded) (17/671, 2.53%), tumor (17/671, 2.53%), cyst (12/671, 1.79%), syringomyelus (4/671, 0.60%), dural ectasia (1/671, 0.15%), and undiagnosed cord MRI hyperintensity (1/671, 0.15%). Many CS patients had two or more intraspinal abnormalities. The manifestations of intraspinal abnormalities are shown in Table 2.

**Table 2:** Manifestations of intraspinal abnormalities

Intraspinal abnormality	Number of patients	Percent
Diastematomyelia	306	45.60
Syringomyelia	273	40.69
Tethered cord	190	28.32
Low conus	58	8.64
Intraspinal mass	39	5.81
Chiari malformation	32	4.77
Fatty filum	27	4.02
Spina bifida (occulta excluded)	17	2.53
Tumor	17	2.53
Cyst	12	1.79
Syringomyelus	4	0.60
Dural ectasia	1	0.15
Undiagnosed cord MRI hyperintensity	1	0.15

**Fig. 3:**
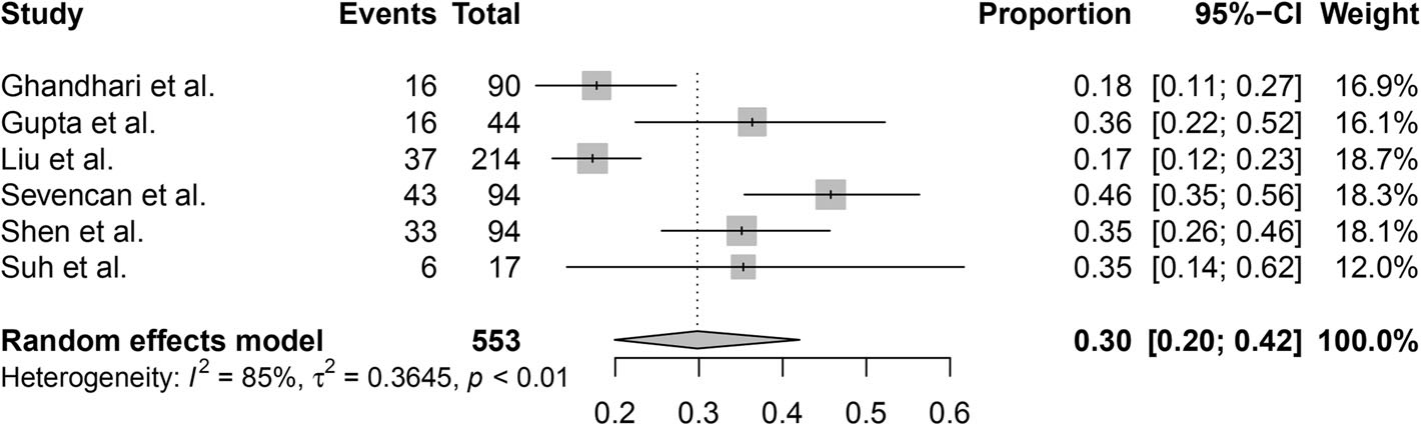
The pooled incidence of intraspinal abnormalities in male patients with congenital scoliosis

**Fig. 4:**
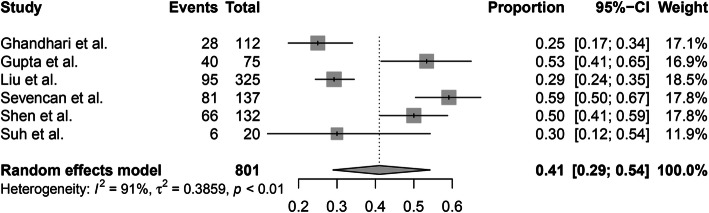
The pooled incidence of intraspinal abnormalities in female patients with congenital scoliosis

The incidence of intraspinal abnormalities in males and females could be pooled from 6 of 10 studies. A random-effects model was used due to the high level of heterogeneity (*I*^2^ = 85%), and the pooled incidence of intraspinal abnormalities in males was 30% (95% CI, 20–42%) (Fig. 3). A random-effects model was used because of the high level of heterogeneity (*I*^2^ = 91%), and the pooled incidence of intraspinal abnormalities in females was 41% (95% CI, 29–54%) (Fig. 4). There were no significant differences in the incidence of intraspinal abnormalities between males and females (*P* > 0.05).

**Fig. 5:**
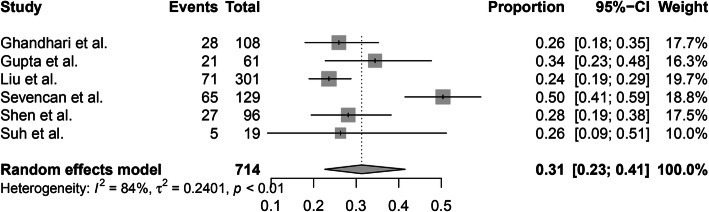
The pooled incidence of intraspinal abnormalities in congenital scoliosis patients with a failure of formation

**Fig. 6:**
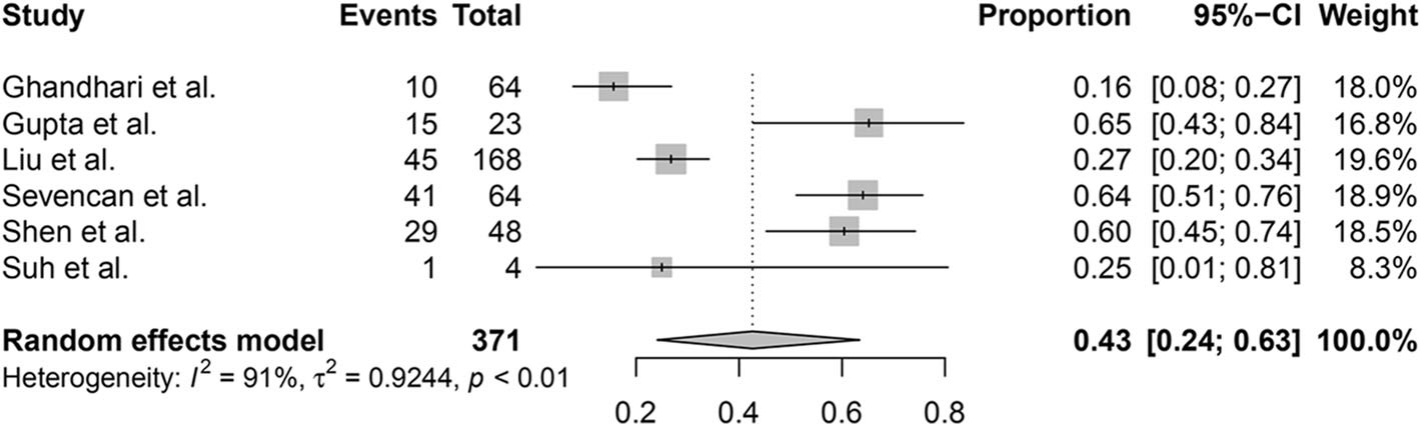
The pooled incidence of intraspinal abnormalities in congenital scoliosis patients with a failure of segmentation

**Fig. 7:**
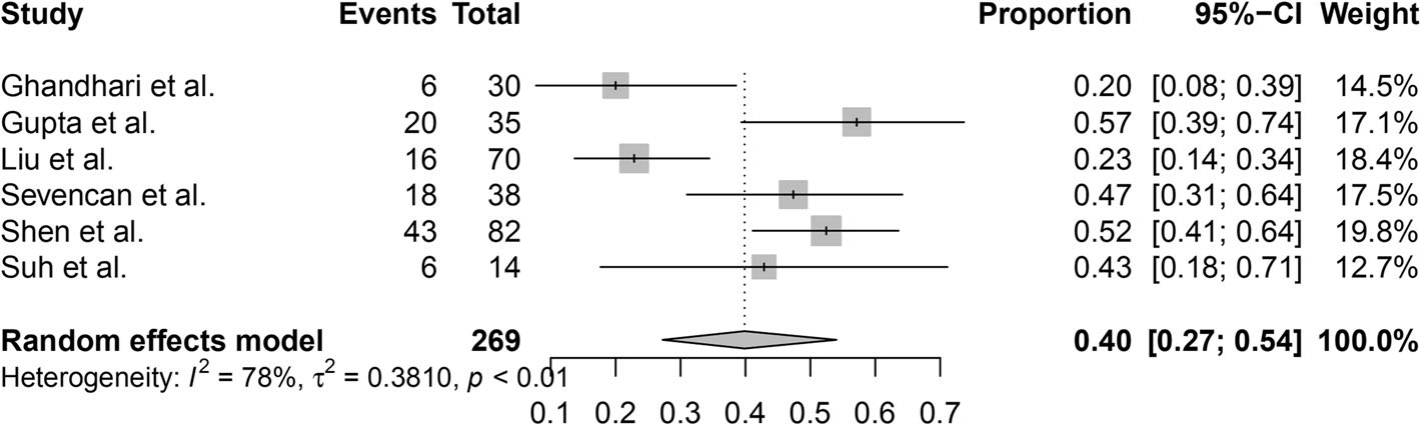
The pooled incidence of intraspinal abnormalities in congenital scoliosis patients with mixed abnormalities

In addition, 6 of 10 studies reported the incidence of intraspinal abnormalities in patients with different CS types. A random-effects model was used because of the high level of heterogeneity (*I*^2^ = 84%), and the pooled incidence of intraspinal abnormalities in patients with a failure of formation was 31% (95% CI, 23–41%) (Fig. 5). A random-effects model was used due to the high level of heterogeneity (*I*^2^ = 91%), and the pooled incidence of intraspinal abnormalities in patients with a failure of segmentation was 43% (95% CI, 24–63%) (Fig. 6). The random-effects model was used due to the high level of heterogeneity (*I*^2^ = 78%), and the pooled incidence of intraspinal abnormalities in patients with mixed abnormalities was 40% (95% CI, 27–54%) (Fig. 7). There were no significant differences in the incidence of intraspinal abnormalities across patients with different CS types (*P* > 0.05).

## Discussion

In the current study, ten studies published from 2001 to 2009 were included, with a total of 1863 patients with CS. To the best of our knowledge, this was the largest study to investigate the incidence of intraspinal abnormalities in patients with CS, and we concluded that the overall incidence of intraspinal abnormalities in patients with CS was 37%.

The intraspinal abnormalities observed in our study included diastematomyelia (45.60%), syringomyelia (40.69%), tethered cord (28.32%), low conus (8.64%), intraspinal mass (5.81%), Chiari malformation (4.77%), fatty filum (4.02%), spina bifida (occulta excluded) (2.53%), tumor (2.53%), cyst (1.79%), syringomyelus (0.60%), dural ectasia (0.15%), and undiagnosed cord MRI hyperintensity (0.15%). Patients with CS can have two or more intraspinal abnormalities. Diastematomyelia was found to be the most common intraspinal abnormality, as it was detected in 45.60% of CS patients with intraspinal abnormalities. This result was different from those in previous studies, which reported that tethered cord was the most common intraspinal abnormality [20, 23].

Sex differences were implicated in the curve progression of scoliosis [30]. However, there is no consensus on the sex differences in the incidence of intraspinal abnormalities. Faloon et al. [31] concluded that there was no difference between males and females in the proportion of adolescent idiopathic scoliosis cases with neuraxial abnormalities. Sevencan et al. [16] reported that in CS patients, females had a significantly higher incidence of intraspinal abnormalities than males. In the present study, we concluded that the incidence of intraspinal abnormalities in males and females were 30% and 41%, respectively. However, there was no significant difference in the incidence of intraspinal abnormalities between males and females (*P* > 0.05).

Currently, it is not clear whether CS types affect the incidence of intraspinal abnormalities in patients with CS. Xue et al. [32] conducted a study with a total population of 118 and found that the incidence of intraspinal abnormalities is higher in CS patients with mixed abnormalities. Liu et al. [1] found that the incidence of intraspinal abnormalities were similar in patients with a failure of formation, a failure of segmentation, and mixed abnormalities. Shen et al. [19] reported that the incidence of intraspinal abnormalities in patients with a failure of segmentation or mixed abnormalities was significantly higher than that in patients with a failure of formation. Contrary to these findings, we found no significant difference in the incidence of intraspinal abnormalities across patients with different CS types (*P* > 0.05). Interestingly, the number of patients with a failure of formation was higher than that of patients with other types of abnormalities, but the corresponding incidence rates of intraspinal abnormalities were similar.

There are some limitations of our research study. Initially, there was a high degree of heterogeneity among the studies. The high degree of heterogeneity may be caused by varieties of participants. In addition, many studies did not report details regarding the age of the CS patients, other corresponding organ defects, and other potential factors that can influence the incidence of intraspinal abnormalities. These data were not explicitly reported, limiting the scope of our research. Finally, this current meta-analysis excluded the article that was not published in English. Therefore, it may be have language restrictions.

## Conclusions

In conclusion, this meta-analysis revealed that the overall incidence of intraspinal abnormalities found by MRI in CS patients was 37%. Diastematomyelia was the most common intraspinal abnormality. Sex and CS type were not factors that affected the incidence of intraspinal abnormalities in CS patients.

## Supplementary information

**Additional file 1:.** PubMed search strategy (CSV 266 bytes)

**Additional file 2:.** EMBASE search strategy (XLS 19 kb)

**Additional file 3:.** Cochrane search strategy

## Data Availability

As a meta-analysis, all raw data of this study are extracted from ten included studies. The datasets supporting the conclusions of this article are available in the ten included studies [1, 16, 19–25, 28].

## Abbreviations

CS: Congenital scoliosis
MRI: Magnetic resonance imaging
MINORS: Methodological Index for Non-Randomized Studies
CI: Confidence interval

## References

[1] Liu YT (2011). A retrospective study of congenital scoliosis and associated cardiac and intraspinal abnormities in a Chinese population. European Spine Journal.

[2] Pahys JM, Guille JT (2018). What's new in congenital scoliosis?. J Pediatr Orthop.

[3] SHANDS AJ, EISBERG HB (1955). The incidence of scoliosis in the state of Delaware; a study of 50,000 minifilms of the chest made during a survey for tuberculosis. J Bone Joint Surg Am.

[4] Giampietro PF (2003). Congenital and idiopathic scoliosis: clinical and genetic aspects. Clin Med Res.

[5] Giampietro PF (2013). Clinical, genetic and environmental factors associated with congenital vertebral malformations. Mol Syndromol.

[6] Hedequist D, Emans J (2007). Congenital scoliosis: a review and update. J Pediatr Orthop.

[7] Basu PS, Elsebaie H, Noordeen MH (2002). Congenital spinal deformity: a comprehensive assessment at presentation. Spine (Phila Pa 1976).

[8] Blake NS, Lynch AS, Dowling FE (1986). Spinal cord abnormalities in congenital scoliosis. Ann Radiol (Paris).

[9] Beals RK, Robbins JR, Rolfe B (1993). Anomalies associated with vertebral malformations. Spine (Phila Pa 1976).

[10] Mohanty S, Kumar N (2000). Patterns of presentation of congenital scoliosis. J Orthop Surg (Hong Kong).

[11] Bollini G (2010). Congenital abnormalities associated with hemivertebrae in relation to hemivertebrae location. Journal of Pediatric Orthopaedics Part B.

[12] Hamzaoglu A (2007). Simultaneous surgical treatment in congenital scoliosis and/or kyphosis associated with intraspinal abnormalities. Spine (Phila Pa 1976).

[13] Noordeen MH, Taylor BA, Edgar MA (1994). Syringomyelia. A potential risk factor in scoliosis surgery. Spine (Phila Pa 1976).

[14] Prahinski JR (2000). Occult intraspinal anomalies in congenital scoliosis. J Pediatr Orthop.

[15] McMaster MJ (1984). Occult intraspinal anomalies and congenital scoliosis. J Bone Joint Surg Am.

[16] Sevencan A (2019). The incidence and interrelationship of concomitant anomalies in congenital scoliosis. Turk Neurosurg.

[17] Peer S (1994). The value of MRI in the preoperative assessment of scoliosis. Orthopade.

[18] Bradford DS, Heithoff KB, Cohen M (1991). Intraspinal abnormalities and congenital spine deformities: a radiographic and MRI study. J Pediatr Orthop.

[19] Shen J (2013). Abnormalities associated with congenital scoliosis: a retrospective study of 226 Chinese surgical cases. Spine (Phila Pa 1976).

[20] Gupta N (2016). Vertebral and intraspinal anomalies in Indian population with congenital scoliosis: a study of 119 consecutive patients. Asian Spine J.

[21] Erfani MA (2007). Occult intraspinal abnormalities and congenital scoliosis. Journal of Research in Medical Sciences.

[22] Belmont PJ (2004). Intraspinal anomalies associated with isolated congenital hemivertebra: the role of routine magnetic resonance imaging. J Bone Joint Surg Am.

[23] Furdock R, Brouillet K, Luhmann SJ (2019). Organ system anomalies associated with congenital scoliosis: a retrospective study of 305 patients. Journal of Pediatric Orthopaedics.

[24] Ghandhari H (2015). Vertebral, rib, and intraspinal anomalies in congenital scoliosis: a study on 202 Caucasians. European Spine Journal.

[25] Hou D (2018). Abnormalities associated with congenital scoliosis in high-altitude geographic regions. International Orthopaedics.

[26] Mik G (2009). Diminished spinal cord size associated with congenital scoliosis of the thoracic spine. J Bone Joint Surg Am.

[27] Rajasekaran S (2010). Intraspinal anomalies in scoliosis: an MRI analysis of 177 consecutive scoliosis patients. Indian J Orthop.

[28] Suh SW (2001). Evaluating congenital spine deformities for intraspinal anomalies with magnetic resonance imaging. Journal of Pediatric Orthopaedics.

[29] Slim K (2003). Methodological index for non-randomized studies (minors): development and validation of a new instrument. ANZ J Surg.

[30] Janicki JA, Alman B (2007). Scoliosis: Review of diagnosis and treatment. Paediatr Child Health.

[31] Faloon M (2018). Incidence of neuraxial abnormalities is approximately 8% among patients with adolescent idiopathic scoliosis: a meta-analysis. Clin Orthop Relat Res.

[32] Xue XH (2013). Analysis of ribs and intraspinal anomalies in congenital scoliosis. Zhonghua Wai Ke Za Zhi.

